# Metabolomics Monitoring of Treatment Response to Brain Tumor Immunotherapy

**DOI:** 10.3389/fonc.2021.691246

**Published:** 2021-06-03

**Authors:** Farhad Dastmalchi, Loic P. Deleyrolle, Aida Karachi, Duane A. Mitchell, Maryam Rahman

**Affiliations:** Department of Neurosurgery, Preston A. Wells, Jr. Center for Brain Tumor Therapy, University of Florida, Gainesville, FL, United States

**Keywords:** metabolic signature, metabolomics monitoring, biomarker, cancer, brain tumor, immunotherapy

## Abstract

Immunotherapy has revolutionized care for many solid tissue malignancies, and is being investigated for efficacy in the treatment of malignant brain tumors. Identifying a non-invasive monitoring technique such as metabolomics monitoring to predict patient response to immunotherapy has the potential to simplify treatment decision-making and to ensure therapy is tailored based on early patient response. Metabolomic analysis of peripheral immune response is feasible due to large metabolic shifts that immune cells undergo when activated. The utility of this approach is under investigation. In this review, we discuss the metabolic changes induced during activation of an immune response, and the role of metabolic profiling to monitor immune responses in the context of immunotherapy for malignant brain tumors. This review provides original insights into how metabolomics monitoring could have an important impact in the field of tumor immunotherapy if achievable.

## Introduction

Immunotherapy for cancer has gained increasing enthusiasm with certain high-profile examples of success in traditionally resistant solid tissue tumors (1–5). Immunotherapy can be delivered with various platforms and all of them lead to anti-tumor adaptive immune responses systemically and within the tumor microenvironment. These strategies include but not limited to dendritic cell (DC) vaccines (6, 7), peptide vaccines (EGFRVIII and heat shock protein) (8), chimeric antigen receptor (CAR)-T cells (9), use of hematopoietic stem cells (10), and of course immune checkpoint inhibitors [e.g. programmed cell death protein 1 (PD-1)/programmed death-ligand 1 (PD-L1) (11, 12) and CTLA-4 (13)]. However, the efficacy of these therapies relies on the ability to induce an adaptive immune response. Early determination of an effective immune response during the treatment course would allow identification of non-responders prior to tumor progression and an early change in treatment. This approach would prevent patients from receiving ineffective treatment, and potentially result in better clinical outcomes.

Several methods have been developed to evaluate adaptive antigen responses including delayed-type hypersensitivity (14, 15), tetramer analysis (16), ELISA (15, 17) (enzyme-linked immunosorbent assay) for measuring bulk cytokine production, ELISPOT (18) (enzyme-linked immune absorbent spot assay) for measuring individual cytokine-producing T cells, flow cytometry-based (19) analysis of cytokine expression, and PCR (20) (polymerase chain reaction) based detection of T-cell receptor gene usage or cytokine production (21). Limitations to these approaches are the magnitude of T cell proliferation necessary to measure response, and measurement of only antigen specific T cell responses. Moreover, these measures have not been shown to have robust and reproducible correlations with patient outcomes (16, 21–23).

An alternative to traditional immune assays is the use of metabolomics to assess the dynamic immune related changes that ensue after immunotherapy. In the last decade, most cancer-related metabolomic studies focused on the tumor microenvironment for use as a diagnostic or prognostic tool (24, 25). Using metabolomic profiling to evaluate immune responses is a novel area of cancer research with the potential to develop methods for measurement of global dynamic changes that may correlate with treatment response or overall outcome. This review will discuss metabolomics methodology, changes in the metabolism of immune cell subsets that can be measured in the context of malignant brain tumors, and the use of metabolomics to evaluate patients receiving immunotherapy for brain tumors.

## Metabolomics Methodology

Metabolites are most commonly measured using mass spectrometry (MS) or nuclear magnetic resonance spectroscopy (NMR). Less common techniques include Fourier transform infrared spectroscopy (FT-IR), ultraviolet-visible spectroscopy (UV) and Raman spectroscopy.

- Nuclear magnetic resonance spectroscopy (NMR)
  NMR is a spectroscopic technique that uses spin properties of the nucleus of atoms to detect metabolites. NMR is fast and ideal for screening. It requires minimum sample preparation and generates structural information (25). NMR has been used in brain tumor studies to identify the tumor related and treatment related metabolic shifts in patients body fluids including urine and blood (26–28).
- Gas chromatography/mass spectrometry (GC/MS)
  GC/MS is more sensitive compared to NMR, but is also more time intensive and expensive. GC/MS allows for detection of small concentrations. GC/MS is often used for more detailed analysis after initial screening with NMR (29). GC/MS has been used in brain tumor studies to identify the tumor-associated metabolites in serum (30), Cerebrospinal fluid (CSF) (31), extracellular fluid (32) and tumor (33) samples.
- Liquid chromatography-mass spectrometry (LC-MS)
  LC is the most versatile separation method. LC-MS can separate compounds in a broad spectrum of polarity with less hassle in sample preparation. Liquid chromatography is used to separate metabolites to overcome problems associated with direct mass spectrometry analysis of complex biological samples. LC-MS was used for metabolomics analysis in brain tumors to phenotyping the glioma tumors (34).
- Seahorse XF technology
  Seahorse XF measures dissolved oxygen and proton excretion to calculate rate of mitochondrial respiration and glycolysis. This technology allowing real time functional monitoring of the metabolic profile of cells, represent an innovative tool to interrogate T cell proliferation, activation and phenotype for example (35, 36).
- Sample preparation
  Sample preparation is incredibly important for metabolomics and can impact overall conclusions. In all biological systems, metabolites of a broad spectrum of chemical diversity exist in a variable range of concentrations. A typical biological cell contains about 5000 metabolites at varying concentrations, which can make identification of most of the metabolites challenging (37). Therefore, the quality of sample preparation technique, environment, and quantity of prepared sample may significantly affect the spectrum of the detected metabolome (38). One strategy to improve sample preparation includes sequential extractions and concentrations to favor a particular class of compound that may be of interest (37). Attention to sample preparation is key to identifying the metabolomic changes as these responses may be of small overall magnitude within the host.

## Overview of Cellular Metabolism

**Figure 1:**
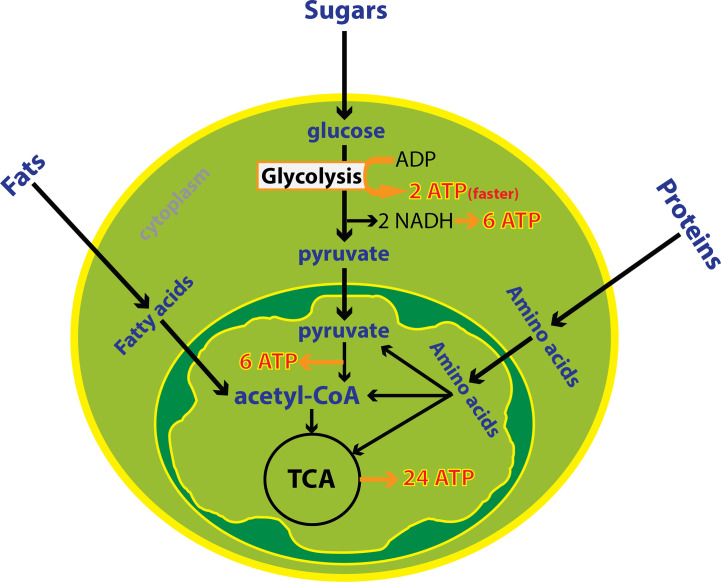
Cytoplasmic and mitochondrial metabolic pathways. Cellular energy is produced through metabolism of fats, sugars and proteins in the mitochondria in the presence of oxygen or the breakdown of glucose in the absence of oxygen in the cytoplasm. Oxidative metabolism in the mitochondria produces more ATP compared to anaerobic metabolism. Despite, glycolysis is less efficient but it is one hundred times faster than oxidative metabolism. In total, 38 ATP is derived from one molecule of glucose including 8 ATP from cytoplasmic metabolism and 30 ATPs from mitochondrial metabolism.

Immune cells undergo large metabolic shifts as they mature and activate during an immune response. These changes can be profound during a robust immune response and can be detected from blood, urine or tumor samples. Cellular energy production is achieved through metabolism of fats, sugars and proteins in the *mitochondria* in the presence of oxygen or through the breakdown of just sugars in the absence of oxygen in the *cytoplasm*. Oxidative metabolism in the mitochondria produces 20 times more ATP compared to anaerobic metabolism (39). The mitochondria uses three enzymatic processes to generate ATP: the tricarboxylic acid (TCA) cycle, oxidative phosphorylation (OXPHOS), and fatty acid-beta oxidation (FAO). Generation of acetyl-CoA through glycolysis and FAO leads to intermediates necessary for the TCA cycle and ultimately OXPHOS. On the other hand, anaerobic glycolysis in the cytoplasm is often utilized by cells that are actively proliferating or acquiring effector function. Glycolysis is characterized by high utilization of glucose and glutamine, and shunting of pyruvate to produce lactate in the cytosol, even in the presence of abundant oxygen. This program requires high nutrient input but also allows metabolic intermediates to be used for biosynthesis. Overall, aerobic glycolysis is less efficient for ATP production than OXPHOS however it is a faster process supporting cell proliferation and activation (40) (**Figure 1**).

Generally, cells have the ability to switch cellular metabolism between oxidative and anaerobic based on their metabolic demands. The metabolic demands of immune cell subsets in circulation and within the tumor microenvironment shift depending on their functional status. Cells important for innate and adaptive immunity produce metabolites that can be detected in the peripheral blood or urine using metabolomic analysis.

## Metabolomics of Innate Immunity

### Tumor-Associated Macrophages

**Figure 2:**
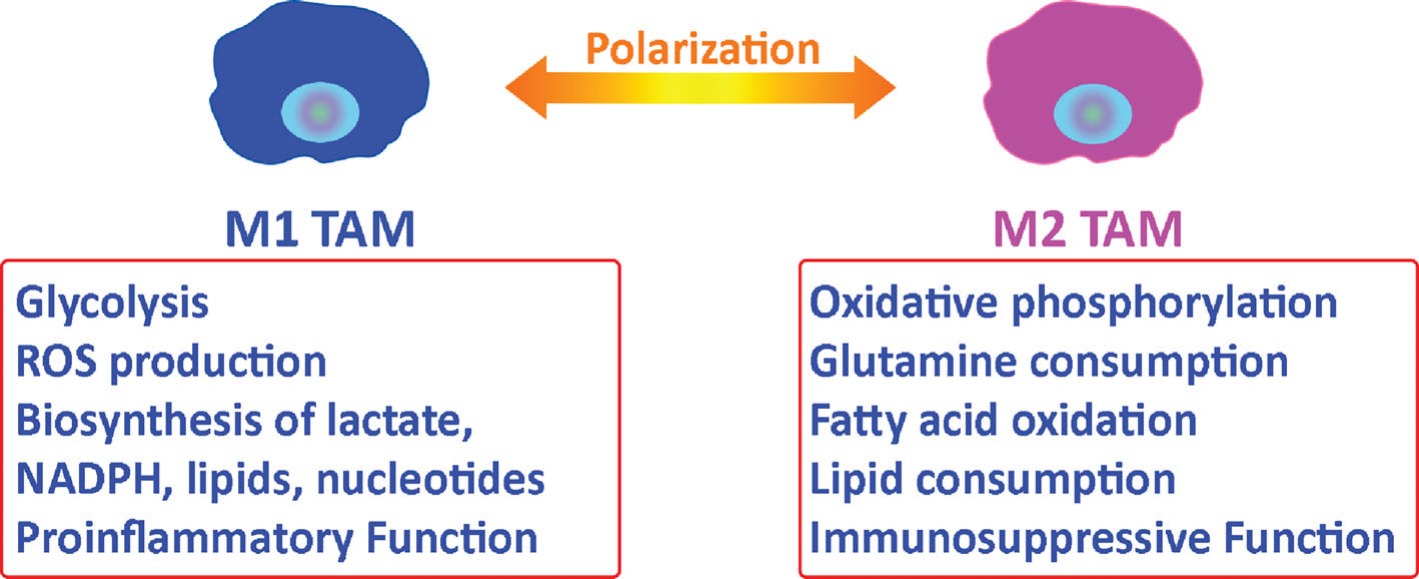
Metabolic relationship between tumor-associated macrophages and tumor cells. Macrophages can be polarized to an M1 or M2 state. M2 tumor-associated macrophages support tumor progression by providing nutritional demands for tumor cells proliferation and suppressing the immune response in the tumor microenvironment.

Tumor-associated macrophages (TAMs) represent the majority of tumor-infiltrating myeloid cells in most solid malignancies and are identified by CD68 (41, 42). TAMs support tumor progression and provide an environment that promotes tumor growth (41, 43). Macrophages can be polarized to an M1 (pro-inflammatory, infection-response) or M2 (anti-inflammatory, tissue-repair) state (44). In general, M1- macrophages are characterized by a glycolytic metabolism with high lactate secretion as well as biosynthesis of NADPH, lipids, and nucleotides. M1 macrophages also vigorously produce reactive oxygen species (ROS) (40) that leads to cytocidal function. Alternatively, M2 macrophages use oxidative metabolism for bioenergetics purposes, which allows for tissue repair (40) (**Figure 2**). M2 macrophages have elevated glutamine and fatty acid consumption. Interestingly, TAMs have alternative metabolic programs including lipid metabolism that results from dysregulated enzymes including acetyl-CoA dehydrogenase medium chain and monoglyceride lipase (45–47). This lipid metabolism is a sign of their metabolic fitness.

The data on the metabolism of tumor-infiltrating TAMs is mixed. In some studies, TAMs utilizing OXPHOS was associated with increased immunosuppression and poor patient outcomes (43). In other studies, mostly in *in vitro* or murine experiments, found that exposure to tumor cells cause TAMs to shift to glycolysis with a more immunosuppressive phenotype. These TAMs secrete lactate, TNF and IL6 (48–50). Moreover, the canonical markers of M1 or M2 activation can be co-expressed by TAMs (43), explaining the conflicting studies on TAM metabolism within the TME. Single-cell RNA sequencing and deconvolution platforms can address these challenges and identify the macrophage clusters (51). However, due to the complexity of macrophage metabolism in patients with cancer, the use of metabolomics to determine macrophage proliferation or effector function has many challenges.

### Natural Killer Cells

Natural killer (NK) cells are cytotoxic innate lymphocytes that play a major role in the primary immune response (52). NK cells are a potential source of interferon-gamma (IFN-γ) production and NK cell effector function is directly related to metabolism (53). NK cells utilize low levels of glycolysis and OXPHOS when they are resting (54), which is enough for IFN-γ production. Stimulation of NKs results in significant increases in the level of both glycolysis and OXPHOS (55, 56) along with an increase in mitochondrial mass (57).

When NKs are activated, they produce pyruvates which do not enter the tricarboxylic acid (TCA) cycle (57). These pyruvates are metabolized to mitochondrial-citrate by entering the citrate–malate shuttle (CMS) (57). CMS is an alternative for TCA in the mitochondria to produce NADH as an energy source for OXPHOS and ATP synthesis. CMS also generates cytosolic NAD+, which is a necessary cofactor to increase the rate of glycolysis (57). NK cells are well-known effector lymphocytes against cancer. However, tumor-associated NKs can be dysfunctional due to metabolic derangements. In a lung cancer model, NKs were found to have increased expression of fructose-1,6-bisphosphatase (FBP1) which regulates gluconeogenesis (58) and inhibits glycolysis. Upregulation of FBP1 in tumor-associated NKs decreased glycolysis and resulted in less cytotoxicity and viability. Therefore, metabolic markers of glycolysis and CMS would be signatures of NK activation after immunotherapy, but may be blunted in patients with tumor associated immune dysfunction.

### Dendritic Cells

Dendritic cells (DCs) are professional antigen presenting cells (APCs) and regulators of innate and adaptive immunity. The presence of DCs in the TME has been shown to increase the efficacy of immune blockade immunotherapy (59) and adoptive T cell therapy (ACT) (60). These findings demonstrate the importance of DCs in the anti-tumor immune response and support the relevance of their monitoring. DCs recognize pathogens through Toll-like receptors (TLRs) (61), retinoic acid-inducible gene I (RIG−I)−like receptors (RLRs), C−type lectins (62) and nucleotide-binding oligomerization domain (NOD)-like receptors (NLRs) (63). Binding to one of the pathogen recognition receptors causes a cascade of signaling pathways that lead to DC metabolic shifts and activation (63). After activation, DCs mature to present antigen to T cells (64). Immature DCs and tolerogenic DCs use catabolism of proteins and triacylglycerols to synthesize fatty and amino acids or intracellular glycogen for OXPHOS (65, 66). As DCs transition to maturity and activation, they switch their metabolism from OXPHOS to glycolysis and lactic fermentation that generate energy. Inhibition of glycolytic metabolism pathway impairs DC maturation and antigen presenting ability, but other functions of DCs such as phagocytosis are not affected by inhibition of glycolysis (66). Similar to NKs, markers of glycolysis could be used to identify maturing and activated DCs.

### Myeloid-Derived Suppressor Cells (MDSCs)

Myeloid cells originate from the bone marrow and when they are found in the brain TME they have profound immunosuppressive functions (67–69). MDSCs inhibit T cell function through three main mechanisms: 1) arginine depletion, 2) reactive oxygen and nitrogen species production and 3) expressing ligands of T cell inhibitory receptors such as programmed death-ligand 1 (PDL-1) (67, 70). Generally, myeloid cells use glycolysis to supply their metabolic demands. However, tumor-associated MDSCs reprogram their metabolism and undergo fatty acid oxidation (FAO) with significantly increased rates of oxygen consumption (67, 69, 71). MDSCs overexpress the lipid uptake receptors such as CD36, Msr1, Fabp5, CD68, Acsl3 and Acsl4 (67, 69, 71). These markers of MDSCs have the potential to serve as biomarkers of MDSC function and can also serve as therapeutic targets (70).

## Metabolomics of Adaptive Immunity

T cells need glucose and amino acids during their life cycle to differentiate, proliferate, and activate (72). Naïve T cells uptake glucose as their main source of carbon and through glycolysis produce pyruvate (72). Naïve T cells that do not actively proliferate and shuttle pyruvate through the tricarboxylic acid (TCA) cycle to generate ATP using OXPHOS. Acetyl-CoA undergoes a series of reactions in TCA cycle to generate citrate (72). Citrate undergoes reactions to produce donor electrons, which pass through the electron transport chain by NADH and FADH2. Finally, these electrons undergo the process of OXPHOS to generate ATP. Once T cells are activated, they rely more heavily on an anaerobic pathway to generate ATP resulting in more lactate as a byproduct to replenish metabolite intermediates (i.e. NAD+) (73, 74). This process is less efficient; only two ATP per molecule of glucose but it is one hundred times faster, thereby serving rapidly proliferating T cells (73, 74).

T cell activation is strongly dependent on nutrient uptake and glucose metabolism (75). Decreased availability of glucose or glutamine dramatically reduces T cell expansion and cytokine production (75). Additionally, effector T cell differentiation is suppressed by decreasing glucose or glutamine (75). The uptake of glucose and glutamine and the rate of production of byproducts of their metabolism (e.g. lactate) are directly correlated with T cell activation and growth (73, 74). T cell activation begins with the engagement of T cell receptor (TCR) and its interaction with APCs. TCRs bind specific antigens by interacting with a short fragment of peptide bound to MHC (major histocompatibility complex) class I/II molecules on the surface of APCs. MHC class I and II present endogenous and exogenous antigens respectively. TCR signaling is initiated upon binding to its ligand triggering a cascade of molecular events initiating differentiation of naïve T cell into effector T cells. TCR activation relies on several known co-stimulatory receptors including CD2, CD28, CD4, CD8, and integrin molecules. CD28 binding to B7-1 or B7-2 on APCs allows for T cell binding. CD28 also generates a co-stimulatory signal in T cells to increase IL-2 production, leading to T cell proliferation. Consequently, CD28 and CD45 phosphorylate the linker for activation of T cells (LAT), which leads to phosphorylation and activation of the TCR-CD3 complex (76, 77).

**Figure 3:**
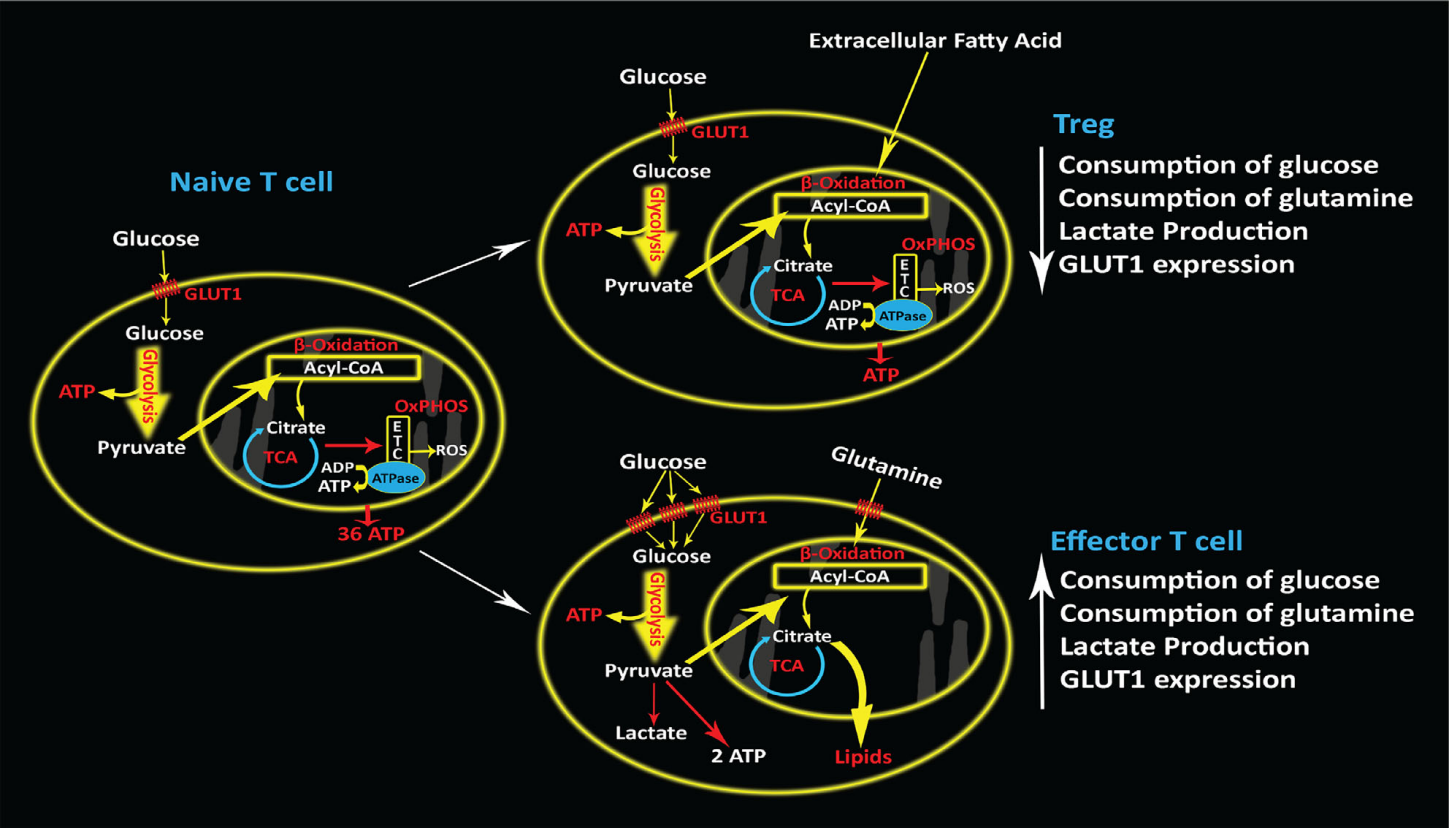
Different T cell subsets and metabolic shifts. Naïve T cells uptake fewer glucose molecules and produce a lower amount of lactate. Tregs uptake more fatty acids compared naïve T cells. Effector T cells express more GLUT1, consume more glucose and glutamine and produce more lactate.

T cell metabolic demands change during the differentiation process. After differentiation, each T cell subset shows different metabolic shifts. Effector T cells which are typically antigen-specific and can cause cytolysis of cells expressing foreign antigens, rely mostly on the glycolytic pathway. The dramatic consumption of glucose and glutamine and high production of lactate has been observed in the early stage of T cells activation in rats (78). This phenomenon is indicated by changes in surface transporters such as GLUT1. CD8+ T effector cells and Th17 cells up-regulate the glycolysis and glucose transporter 1 (GLUT1) (79). Conversely, immunosuppressive regulatory T cells (Tregs) that are a subset of CD4+ T cells have lower needs for glycolysis that leads to less consumption of glucose and glutamine and less production of lactate compared to effector T cells (80). Treg differentiation is not dependent on GLUT1 (81) (**Figure 3**). Alternatively, memory T cells rely on OXPHOS and increase the consumption of the fatty acids to promote this pathway (82, 83). Memory T cells live longer than other subsets and their survival relies on the metabolites which are synthesized through fatty acid oxidation. Also, these metabolites are essential for the memory T cells prompt recall after infection (82, 83). Instead, exhausted T cells express the inhibitory receptors that are known as exhaustion markers such as PD-1, Lag-3 and Tim-3 (84). In the exhaustion process, glucose uptake is reduced, and fatty acid oxidation, and OXPHOS slow down, resulting in overall reduction of metabolite production (84).

**Figure 4:**
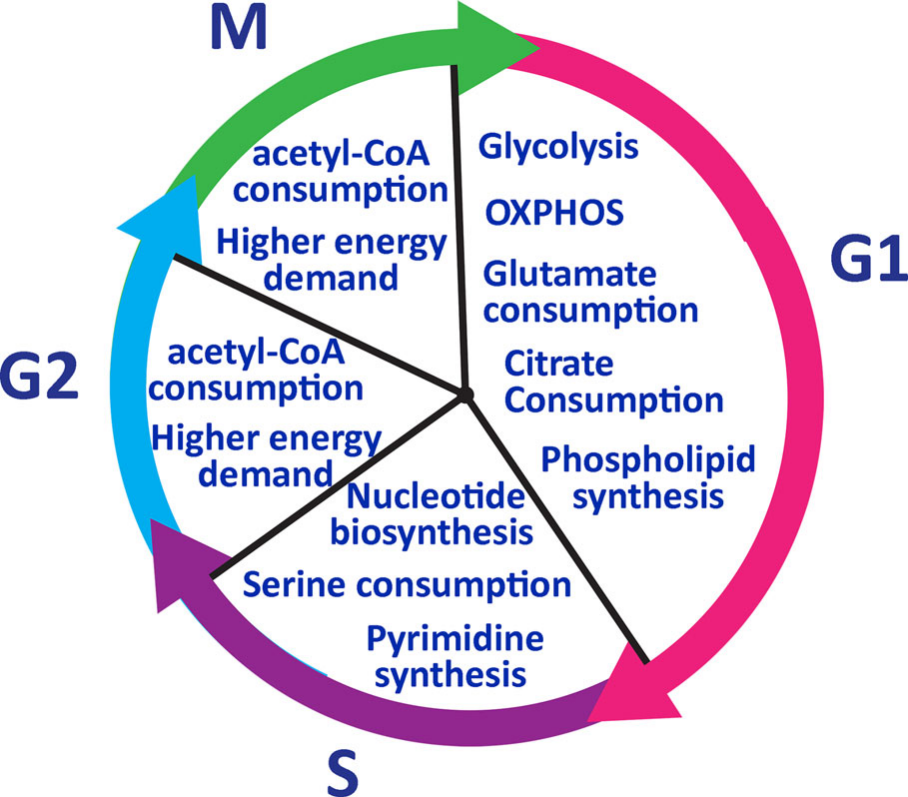
Lymphocyte metabolic pathway during the cell cycle. T cells go through glycolysis metabolic pathway in phase G1 and consume glutamate and citrate to generate phospholipids. In phase S, T cells need to duplicate their genome and serine to synthesize nucleotides. Pyrimidine synthesis occurs only in the S phase and it is required to enter into G2. IN phase G2 and M, T cells need more energy to grow in size and consume acetyl-CoA to generate more ATP.

Transitioning from the resting G_0_ to G_1_, T cells switch temporarily to an oxidative state and mainly utilize OXPHOS related proteins such as mitochondrial ATPase to produce adequate biomass and ATP (74). During G_1_ glutamate is highly taken up and citrate is used for phospholipid synthesis, which is needed for cell growth (74). Citrate is also used to produce cholesterol which is used to create the cell membrane (74). In S phase, T cells require increased nucleotide biosynthesis for genome duplication before undergoing cell division. S phase is also marked by increased serine metabolism for generation of N5, N10-methylenetetrahydrofolate, which are key byproducts in the tetrahydrofolate cycle regulating nucleotide biosynthesis (85). Cells need purines to enter into G_1_ and S phase but pyrimidine synthesis is required only in S phase (86). T cells then pass through phase G_2_ and M to complete the cell division process. For G_2_/M phases, T cells increase in cell size and need more energy (**Figure 4**). Therefore, acetyl-CoA is utilized during these phases. The above-described metabolic shifts can serve as reliable markers of immune responsiveness to treatment. These metabolic changes can indicate the state of host T cells and dominant subsets of T cells after differentiation.

## Clinical Metabolomics and Immunotherapy

As discussed, as immune cell subsets (DCs, NKs and T cells) activate, metabolic shifts to glycolysis are pronounced. These metabolic profile changes have the potential to identify immune responses after treatment with immunotherapy. Metabolomics lends itself to serial analyses as these changes can be detected through NMR analyses of urine or blood samples (87, 88). Therefore, the kinetics of immune responses can be followed. This approach is being tested in human clinical trials. The ATTAC II (NCT02465268) study is a randomized, placebo-controlled trial testing a pp65 CMV RNA DC vaccine platform in patients with newly diagnosed GBM. Part of the analysis includes metabolomic analysis of urine samples over time to correlate with imaging and clinical outcomes.

Most published studies of metabolomics and gliomas revolve around the intra-tumoral metabolic profiles and methods for distinguishing between tumor subtypes and monitoring for recurrence. There are limited studies of the use of metabolomics in the prediction of human patient’s clinical outcomes with brain tumors by using blood samples. A list of several metabolomics studies with a different type of samples and analytical techniques in brain tumor is provided (**Table 1**). In one study, plasma samples were collected from 70 glioma patients with grade III and grade IV (28). NMR spectra of collected plasma samples were analyzed to identify the metabolomics changes associated with glioma in comparison with healthy subject samples as the control group (28). Twenty metabolites were identified, which are related to the presence of glioma. Glioma was associated with a lower level of isoleucine, leucine, valine, lactate, alanine, glycoprotein, glutamate, citrate, creatine, Myo-inositol, choline, tyrosine, phenylalanine, 1-methylhistidine, α-glucose, β-glucose (28). And the higher concentration of very-low-density lipoprotein (VLDL), low-density lipoprotein (LDL), unsaturated lipid, and pyruvate were identified with a significant correlation to the presence of glioma (28). Metabolomic analysis has also been utilized to differentiate glioma grade. Plasma samples were collected from 87 glioma patients and liquid chromatography triple quadrupole mass spectrometry (LC-QQQ-MS) was used to analyze the metabolomics differences which is applicable as host biomarker candidates to classify glioma in patients (34). Five plasma metabolites significantly differed between high grade and low-grade gliomas including uridine, uracil and ornithine which increased in high-grade gliomas, and N-acetylputrescine and trimethylamine-N-oxide (TMAO) which decreased in high-grade gliomas (34). Of note, TMAO is reduced in patients with IDH1 mutation (34). Arginine/proline metabolism was the pathway with the most significant impact (34). In another study, serum samples were used to identify the metabolomics shifts during radiotherapy in glioma patients (30). Serum samples collected from 11 patients in the initial phase of radiotherapy and serum metabolites were identified by using gas-chromatographic- time-of-flight-mass spectroscopy (30). Patients underwent radiotherapy within 2 to 5 days post-surgery and fasting serum samples were collected just before the first radiotherapy session and at days 1, 2 and 5 after radiotherapy fraction (30). A total of 84 serum metabolites differed significantly in the samples after radiotherapy in comparison to before radiotherapy samples as control samples. Among those metabolites, sixteen metabolites increased after radiotherapy while sixty-eight metabolites decreased after radiotherapy in comparison with before treatment samples (30). Citric acid and dehydroascorbic acid dimer were the top metabolites which showed increased level in serum after treatment and ornithine, tyrosine, glutamine, creatinine and glyceric acid were the top significant metabolites that decreased in serum after radiotherapy (30). Clinically relevant metabolomics signatures were studied in other malignancy with brain metastasis, in melanoma and renal cell carcinoma patients treated with nivolumab (PD-1 inhibitor), blood samples were analyzed with LC-MS. Samples from two phase I trials including 78 patients with advanced melanoma and 91 patients with metastatic renal cell carcinoma (RCC) as well as samples from a large randomized phase III trial in which 394 RCC patients received nivolumab and 349 received everolimus. Post-treatment serum samples were compared to pre-treatment (baseline) serum samples. Kynurenine, which is a product of tryptophan catabolism, was the most significant metabolic difference between groups (96). Kynurenine/tryptophan ratios correlated with worse overall survival for patients treated with nivolumab. The kynurenine pathway (KP) breaks down tryptophan leading to production of NAD+. In the presence of pro-inflammatory cytokines, KP is induced by activation of its first enzyme, indoleamine 2,3-dioxygenase (IDO-1) (97). Increased IDO-1 activity (as reflected by higher K/T ratios) is known to suppress the T-cell mediated response. In disease states where K/T ratio is increased, it is thought that there is T cell-mediated response suppression (97). Glioma patients with Isocitrate dehydrogenase 1 (IDH1) mutation have significantly prolonged median survival in comparison to glioma patients with wild-type IDH1 (98). Previously it was shown that glioma tumor cells with IDH1 mutation highly produce 2-hydroxyglutarate (2HG) and considered as a biomarker for IDH1 mutation (99). Therefore, *in vivo* detection of 2HG is critical for the prediction of clinical outcomes. In one study, metabolic changes were studied in gliomas patients with IDH1 mutation (89). Surgical resection samples were obtained from ten glioma patients with grades II-IV including IDH1 positive and negative. Samples compared with U87 glioblastoma cells which overexpress IDH1. Metabolomics analysis through Capillary electrophoresis time-of-flight mass spectrometry (CE-TOFMS) revealed that levels of D-2-hydroxyglutarate (D-2HG) were significantly increased in the glioma patients with an IDH1 mutation (89). It is shown that T cells import extracellular 2HG which is exported by IDH mutant glioma cells, resulting in the suppression of T cell activation and penetration in TME (100, 101). Moreover, it is shown that 2HG effectively blocks an ATP-dependent T cell receptor (TCR) signaling pathway, which results in suppression of T cell proliferation and function (100). Despite the immune suppression role of IDH1 mutation, it is still unknown why patients with IDH-mutant glioma have prolonged overall survival and better clinical outcome (100, 101). Therefore, it is important to monitor 2HG as the direct metabolite of IDH1 mutant glioma along with metabolomics monitoring of immune cells in glioma patients.

**Table 1:** *In vivo* metabolomics studies in brain tumor patients.

Author & year	Patients#	Samples	Metabolomics analytical method	Conditions	Identified metabolites	Result
Yu, 2020 (33)	66	Tumor tissue	GC-MS & LC-MS	Newly diagnosed gliomas	Acylcarnitine & LPE	Short-chain acylcarnitines level were increased, whereas lysophosphatidylethanolamines (LPEs) were decreased in high-grade gliomas
						
Miyata, 2019 (89)	10	Tumor tissue	Capillary electrophoresis time-of-flight mass spectrometry (CE-TOFMS)	Newly diagnosed glioma patients (grades II–IV)	D-2-hydroxyglutarate (D-2HG)	In glioma patients with IDH1 mutation, D-2HG levels were significantly increased D-2HG inhibited α-keto acid transaminase, which leads to inhibition of 2OG production and inhibition of the TCA cycle
						
Kelimu, 2016 (28)	70	Plasma	NMR	Grade III and grade IV glioma	Isoleucine, leucine, valine, lactate, alanine, glycoprotein, glutamate, citrate, creatine, myo-inositol, choline, tyrosine, phenylalanine, 1-methylhistidine, α-glucose, β-glucose, lipoprotein, unsaturated lipid, and pyruvate	20 metabolites were identified, which are related to the presence of glioma Glioma were associated with lower level of isoleucine, leucine, valine, lactate, alanine, glycoprotein, glutamate, citrate, creatine, myo-inositol, choline, tyrosine, phenylalanine, 1-methylhistidine, α-glucose, β-glucose Glioma were associated with higher level of very low density lipoprotein, low density lipoprotein (LDL), unsaturated lipid, and pyruvate
						
Zhao, 2016 (34)	87	Plasma	Liquid chromatography triple quadrupole mass spectrometry (LC-QQQ-MS)	Glioma grade (high & low), GBM, malignant gliomas, and IDH mutation status glioma	Uracil, arginine, lactate, cystamine, and ornithine, • N-acetylputrescine and trimethylamine-N-oxide (TMAO)	Five metabolites including uracil, arginine, lactate, cystamine, and ornithine, significantly differed between high and low grade glioma patients Uridine (P = 3.76 × 10−4, q = 0.015) and ornithine (P = 9.36 × 10−4, q = 0.038) were identified which differed between GBM and malignant glioma patients N-acetylputrescine (P = 9.12 × 10−4, q = 0.036) and trimethylamine-N-oxide (TMAO) (P = 0.006, q = 0.043) were identified which differed between IDH mutation positive and negative tumors
						
Mörén, 2016 (30)	11	Serum	Gas-chromatographic- time-of-flight-mass spectroscopy (GC-TOFMS)	High grade glioma	Myo-inositol, creatinine, urea and citric acid	Concentration of 68 metabolites were decreased following radiotherapy while 16 metabolites were decreased after radiotherapy Myo-inositol, creatinine, and urea were the main metabolites which decreased during the radiotherapy Citric acid increased during the radiotherapy
						
Wilson, 2015 (90)	35	Tumor tissue	Magnetic resonance spectroscop (MRS)	Medulloblastoma	Creatine, glutamate and glycine	Creatine, glutamate and glycine associated with survival (p<0.01)
						
Elkhaled, 2014 (91)	126	Tumor tissue	^1^H HR-MAS spectroscopy	New or recurrent gliomas of grades II–IV	MI, tCho, tGSH and 2HG	Increased ratio of MI/tCho associated with grade II glioma Decrease in MI and increase in tGSH and 2HG indicates transformation from grade II to grade III or IV glioma
						
Nakamizo, 2013 (31)	32	Cerebrospinal fluid (CSF)	GC/MS	Intracranial glial tumors	Citric, isocitric acid & lactic acid	The citric and isocitric acid levels were significantly higher in the glioblastoma (GBM) than in the grades I–II and III glioma The CSF levels of the citric, isocitric, and lactic acids were significantly higher in grade I–III gliomas with mutant IDH than in those with wild-type IDH.
						
Andronesi, 2012 (92)	10	*In vivo* imaging	optimized spectral-editing and two-dimensional (2D) correlation magnetic resonance spectroscopy (MRS)	Glioma patient with IDH1 mutation	2-hydroxyglutarate (2HG)	2HG detected non-invasively in glioma patients with IDH1 mutation two-dimensional (2D) correlation magnetic resonance spectroscopy (MRS) was capable to detect 2HG in vivo.
						
Locasale, 2012 (93)	10	CSF	Targeted mass-spectrometry	Malignant gliomas	Biotin, glucono.d-lactone, dihydroorotate, orotate, 2,3-dihydroxybenzoic acid, Indole.3-carboxylic acid, etc.	39 metabolites significantly changed in the CSF of the malignant gliomas *vs*. the control samples (p < 0.05) The identified metabolites originate from several metabolic pathways such as amino acid, lipid, pyrimidine, and central carbon metabolism
						
Wibom, 2009 (32)	11	Extracellular fluid intracranially	Gas chromatography-time-of-flight mass spectrometry (GC-TOF MS)	High-grade glioma	Dihydroxybutanoic acid, Hydroxybutanoic acid, Arabinose, Myo-Inositol, Pentonic acid, etc.	67 metabolites were identified There were distinct metabolic differences between the intracranially collected samples from tumor and the brain adjacent to tumor (BAT) region There were the systematic metabolic changes induced by radiotherapy treatment among both tumor and BAT samples
						
Marcus, 2007 (94)	76	*In vivo* imaging	Proton magnetic resonance spectroscopic imaging (MRSI)	Pediatric CNS tumors	choline-containing compounds (Cho)	Cho + 0.1L was the only independent predictor of survival (likelihood ratio test = 10.27, P<0.001; Cox regression, P=0.004) Accuracy and specificity for Cho + 0.1L were 80% and 86%, respectively
						
Albers, 2004 (95)	8	*In vivo* imaging	Proton‐decoupled 31P and 1H MRS	Newly diagnosed, untreated pediatric brain tumors	PE/GPE, PC/GPC, Choline & creatine	The significant increased ratios of phosphoethanolamine to glycerophosphoethanolamine (PE/GPE) and phosphocholine to glycerophosphocholine (PC/GPC) were associated with primitive neuroectodermal tumors (PNET) (16.30 ± 5.73 and 2.97 ± 0.93) when compared with controls (3.42 ± 1.62, P < 0.0001 and 0.45 ± 0.13, P < 0.0001) and with other tumors (3.93 ± 3.42, P < 0.001 and 0.65 ± 0.30, P < 0.0001). Choline significantly increased (4.78 ± 3.33 versus 1.73 ± 0.56 mmol/kg, P < 0.05), and creatine decreased in tumors (4.89 ± 1.83 versus 8.28 ± 1.50 mmol/kg, P < 0.05)

Overall, metabolomics has the potential to measure robust immune cell changes from OXPHOS to glycolysis in multiple compartments in a serial fashion. This approach also has the potential for identifying host immune factors that would prevent effective anti-tumor immunity. This field is still in its infancy and further studies in human patients are necessary to determine if the sensitivity and specificity of these techniques will lend themselves to clinical utility.

## Conclusion

**Figure 5:**
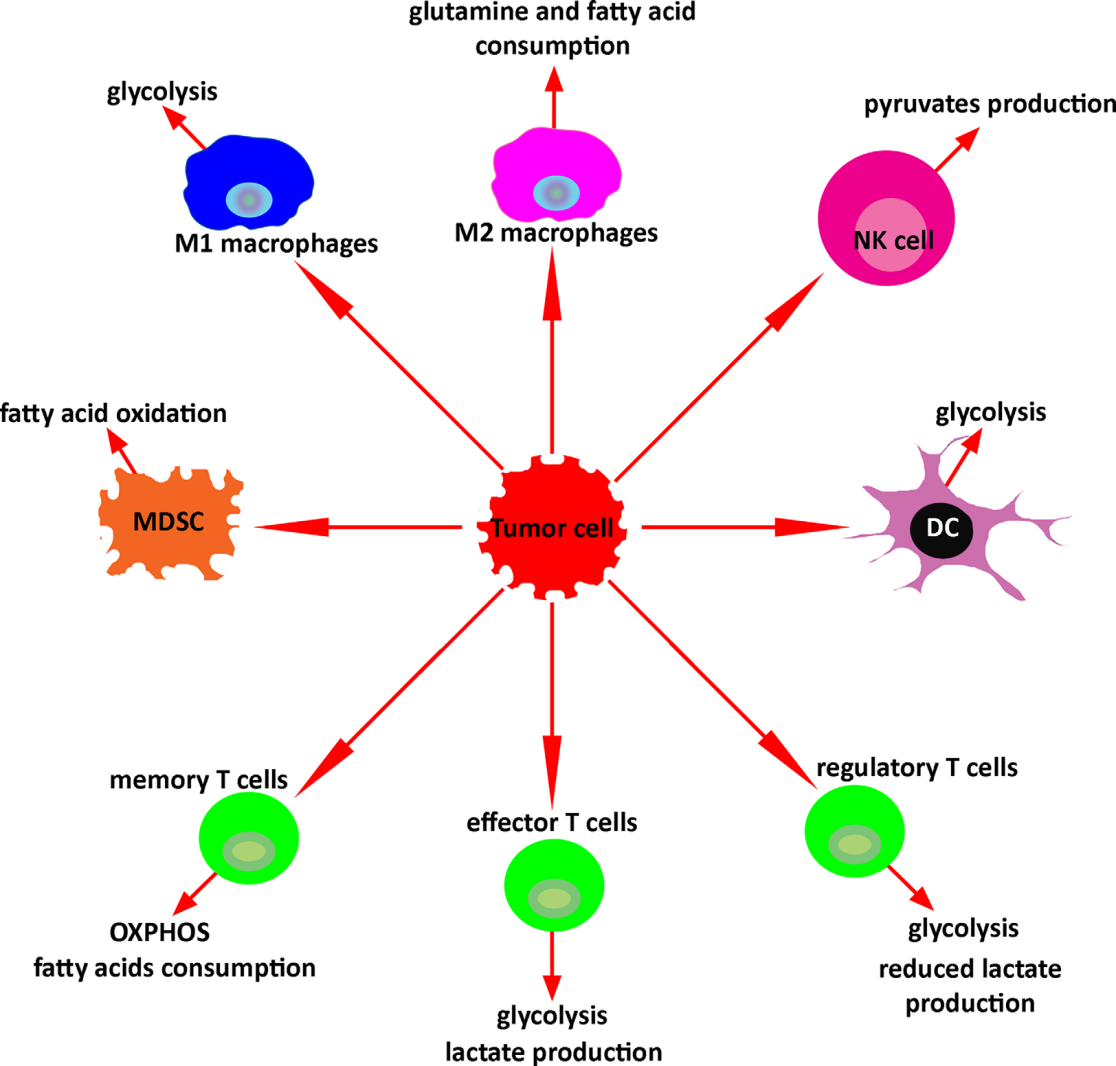
Key metabolism of immune cells in the tumor microenvironment. In response to the tumor microenvironment, immune cells undergo specific metabolic reprogramming regulating their function.

Metabolomic analysis has the potential to study and monitor immune activation after treatment with immunotherapy. In response to the microenvironment, immune cells undergo metabolic reprogramming regulating their function. Upon activation, M1- macrophages activate glycolytic metabolism and secrete lactate, ROS, NADPH, lipids, and nucleotides. Activated M2 macrophages increase glutamine and fatty acid consumption. Stimulated NKs significantly increase both glycolysis and OXPHOS, and activated NKs produce pyruvates as a key metabolite. Tumor-associated MDSCs undergo fatty acid oxidation. Activated DCs switch catabolic metabolism to glycolysis as well. Similarly, effector T cells produce lactate upon activation. Conversely, regulatory T cells produce less lactate. Memory T cells use OXPHOS and increase the consumption of the fatty acids. Exhausted T cells reduce the glucose uptake and decrease the fatty acid oxidations and OXPHOS (**Figure 5**). Altogether, these metabolic changes defining specific immune activities may be used to assess and predict the response to therapy. Such strategies hold great promises and warrant further investigations, especially in the context of patients with brain tumors, and could provide insights into future studies about metabolomics monitoring of immune response in murine glioma models or in patients with different types of malignancies.

## Author Contributions

All authors contributed to the article and approved the submitted version.

## Conflict of Interest

The authors declare that the research was conducted in the absence of any commercial or financial relationships that could be construed as a potential conflict of interest.
